# Commentary: Paracetamol-Induced Glutathione Consumption: Is There a Link With Severe COVID-19 Illness?

**DOI:** 10.3389/fphar.2020.625295

**Published:** 2021-01-26

**Authors:** Sergio Verd, Mateo Verd

**Affiliations:** ^1^ Pediatric Unit, La Vileta Surgery, Department of Primary Care, Palma de Mallorca, Spain; ^2^ Balearic Islands Health Research Institute (IdISBa), Palma de Mallorca, Spain; ^3^ Department of Anesthesiology, Perioperative and Pain Medicine, Son Espases University Hospital, Palma de Mallorca, Spain

**Keywords:** metabolic syndrome, obesity, down syndrome, oxidative stress, hyperthyroidism, glutathione

**A Commentary on**

**Paracetamol-Induced Glutathione Consumption: Is There a Link With Severe COVID-19 Illness?**

*by Sestili, P., and Fimognari, C. (2020). Front. Pharmacol. 11:579944. doi:* *10.3389/fphar.2020.579944*

## Introduction

Glutathione (GSH) is the most important small molecular weight antioxidant produced in the cell. Sestili and Fimognari propose a pathbreaking hypothesis regarding a link between paracetamol-induced GSH depletion and COVID-19. Their study is far from a conclusive hypothesis, it should be considered as a working hypothesis based upon field observations of GSH depletion in some cohorts with COVID-19. The authors support the case that GSH stores are consumed at therapeutic doses of paracetamol, and not only in overdose paracetamol; they remind us that ingestion of therapeutic doses of paracetamol depletes serum antioxidant capacity in healthy volunteers in 14 days, possibly by a reduction in GSH (Nuttall et al., 2003). While Sestili and Fimognari do not offer clinical evidence of GSH being critical in the mortality from COVID-19, they refer to the worldwide positive strong correlation of glutathione S-transferase T1 null genotypes (a predictor of oxidative stress) with COVID-19 mortality rates, unveiled by multivariate analysis (Saadat, 2020).

On the other hand, paracetamol has been shown to have some adverse effects on duration and local severity in some viral infections. Interestingly longer viral shedding was shown in influenza and rhinovirus infections (Table 1; Doran et al., 1989; Graham et al., 1990; Plaisance et al., 2000; Mikaeloff et al., 2008; Ip et al., 2016). Additionally, GSH also plays an important role in cancer development (Ballatori et al., 2009), and melanoma outcomes appear to be influenced by paracetamol intake (Køstner et al., 2015). When 179 patients with metastatic melanoma were retrospectively studied, the authors found a strong positive prognostic effect of high fever. Based on these data, they stopped the routine use of paracetamol to patients with melanoma and renal cell carcinoma treated with IL-2/IFN. However, the association between high fever and improved survival was present only in patients treated without paracetamol. This might be explained by significantly lower body temperatures among the paracetamol-treated patients compared with the patients not receiving paracetamol. However, it cannot be ruled out that it is the use of paracetamol that impairs antitumor immunity and actually has greater impact on patient survival than the body temperature itself. More studies are needed to further clarify this.

**TABLE 1:** Effect of fever suppression by paracetamol on medically attended virus infections.

Author	Disease	Number of participants	Intervention	Outcomes
Doran et al. (1989)	Chickenpox	72	Placebo vs. paracetamol at 80 mg/kg/day	Time to total scabbing was better in the placebo group: 5.6 days (SD: 2.5) vs. 6.7 days (SD: 2.3) (*p* = 0.05)
Graham et al. (1990)	Rhinovirus infection	60	Paracetamol vs. ibuprofen vs. paracetamol vs. placebo	Use of aspirin or acetaminophen was associated with suppression of serum neutralizing antibody response and with increased turbinate oedema and nasal obstruction (*p* < 0.05)
Plaisance et al. (2000)	Influenza A	54	Paracetamol vs. nothing	A correlation was found between paracetamol therapy and duration of illness: 8.8 days on antipyretics vs. 5.3 days without antipyretics (*p* < 0.001)
Mikaeloff et al. (2008)	Chickenpox	386	Paracetamol vs. nothing	The adjusted rate ratio of severe skin or soft tissue complication associated with exposure to paracetamol was 1.5 (95% CI 1.0, 2.2)
Ip et al. (2016)	Influenza	78	Paracetamol vs. placebo	The mean duration of viral shedding in the treatment group was 7.8 days compared to 6.1 days in the placebo group (*p* = 0.02)

CI, confidence interval; SD, standard deviation.

On the following paragraphs we expose the link between low GSH levels, paracetamol administration and severe symptoms of COVID-19.

## Subsections Relevant for the Subject

### Elderly Patients

COVID-19 disproportionately affects old people (Mueller et al., 2020). Changes that occur throughout the ageing process place the elderly population at a greater risk of malnourishment, and inclined to an increased utilization of paracetamol (Nishtala et al., 2015). Both conditions may be associated with GSH stores depletion.

### Down’s Syndrome

Increasing number of studies have recently shown that oxidative stress, evidenced by decreased GSH levels, occurs in down’s syndrome (DS) progression (Garlet et al., 2013). Very recent research estimates a 10-fold increased risk for COVID-19-related death in persons with DS (Clift et al., 2020). It is worthy of our attention that there is evidence of increased paracetamol-glutathione conjugation after administration of 20 mg/kg oral doses of paracetamol to DS patients as compared to controls (Griener et al., 1990).

### Hyperthyroidism

A significant decrease in GSH levels is observed in the hyperthyroid patient (Ali and Sultan, 2011). COVID-19 may be associated with a high risk of autoimmune hyperthyroidism. On the other hand, hyperthyroidism may also be associated with a high risk of severe COVID-19, thyrotoxicosis may trigger severe SARS-CoV-2 infection, in fact serum thyrotropin values are inversely correlated with higher IL-6 (Lania et al., 2020). There are no reports of pediatric patients with confirmed SARS-CoV-2 infection managed with ECMO support in Germany (Armann et al., 2020) or Spain (González Cortés et al., 2020), except an 11 years old girl with Graves’ disease (Ultimahora, 2020). One of the main effects of thyroid hormones in Graves’ disease is an increase in the total consumption of oxygen, which results in increased formation of free radicals, or the occurrence of oxidative stress (Vrca et al., 2004).

### Metabolic Syndrome

The metabolic syndrome (MetS) is a cluster of at least three medical conditions. It is known that GSH decreases dramatically when four MetS factors are present (Butkowski et al., 2017), and that waist circumference is one of the main predictors of low GSH levels among these patients (Awadallah et al., 2019). Medical research has repeatedly concluded that those patients suffering from COVID-19 as well as MetS are at a high risk of developing severe disease. A severe course of COVID-19 is a result of both the direct cytolytic effects of SARS-CoV-2 and the imbalanced production of reactive oxygen species by the cell resulting in extensive inflammation and tissue destruction (Schönrich et al., 2020). GSH not only sweeps away oxygen free radicals, but also regenerates other oxidised antioxidants and takes part in the repair of oxidative stress-damaged biomolecules (Choromańska et al., 2020). It has been reported that erythrocyte GSH was significantly lower in patients who received 100 mg/kg/day of paracetamol compared with patients who received paracetamol at a dose of 50 mg/kg/day and those who did not receive paracetamol (Kozer et al., 2003).

## Discussion

COVID-19 is a new pathological disease, and an increasing number of studies have come to the conclusion that oxidative stress is a major cause of local or systemic tissue damage that leads to severe COVID-19. In this commentary, we have highlighted that DS, MetS, Graves’ disease or ageing are linked to the accumulation of oxidative damage to macromolecules. In addition, we report that therapeutic doses of paracetamol can affect the antioxidant function of GSH.

Current guidelines on the symptomatic management of fever in children agree that the objective of antipyretic therapy is not euthermia but rather to make the child comfortable (Chiappini et al., 2017). Nonetheless, a Cochrane review has concluded that there is insufficient evidence to conclude that paracetamol is superior to placebo in decreassing the child’s discomfort (Meremikwu and Oyo-Ita, 2002). This small size effect was highlighted by the inability of parents from a randomised placebo-controlled trial to correctly guess whether their children had received paracetamol or placebo (Kramer et al., 1991).

In light of the above, to continue the generous use of paracetamol may mean that we are ignoring important messages from research. It may not be worrisome in healthy adults. Conversely, in the midst of a devastating pandemic, it is more important than ever to present satisfactory arguments for prescribing paracetamol in sensitive populations with scarce GSH levels. We have described in detail four serious health conditions associated with the reductions of GSH and the risks of developing severe COVID-19.

We suggest the safety and efficacy of paracetamol should be further studied in COVID-19. Future research could attempt to correlate death rate attributable to COVID-19 with figures of paracetamol daily doses per 1,000 inhabitants (DDD) within countries with similar standard of life.

## Conclusion

In current times, the statement that antipyretics do not cause any lasting damage must be carefully reconsidered. It is cautionary to refrain from prescribing paracetamol to patients with a self-perpetuating inflammatory response. The elderly, as well as patients with DS, hyperthyroidism, or the MetS are eligible for a “minimal effective does for the shortest possible period” approach, similar to the European Medicines Agency (EMA, 2020) current advice for NSAIDs.

## Author Contributions

SV: conceptualization and writing. MV: bibliographic search, revision of the text. All authors contributed to the article and approved the submitted version.

## Conflict of Interest

The authors declare that the research was conducted in the absence of any commercial or financial relationships that could be construed as a potential conflict of interest.
